# Maintaining Confidence in Police Use of Force in Western Liberal Democracies

**DOI:** 10.1007/s41887-022-00076-9

**Published:** 2022-07-13

**Authors:** Neil Basu

**Affiliations:** Metropolitan Police Service, London, UK

**Keywords:** Democracy, Police, Use of force

## Abstract

In a liberal democracy, the police cannot have the power to use force without accountability and transparency. Operational independence to make hard policing decisions should be matched by operational responsibility and willingness to account for that decision. Political and societal pressure can result in over- or under-reaction in the use of hard police tactics. *We are not making decisions based on rigorous evidence and research, so we cannot explain why we use certain tactics and whether they are disproportionate*. The evidence concerning our need for more self-protection and greater force is poor and may affect our judgement. Public confidence requires both better evidence and better communication of that evidence to communities in which police use force with greatest consequence.

## Introduction

Shortly after the invasion of Ukraine, a Russian attempt to recruit local residents to police their own conquered territories was met by an unequivocal and unprintable Ukrainian refusal. That news was a reminder of the value of the UK policing model of policing by consent of those who are policed, with independence from the state. So, too, is the counter-example of the comments of a Rabbi I once heard while standing inside Auschwitz. The Rabbi was reviewing the role of the police in the Holocaust. The German police, and the police in many territories Germany conquered, did not resist Nazi genocide in World War 2.

The lesson of these two cases, for me, is that the separation of powers is of crucial importance. The police must make sure we are not, and are never seen to be, an arm of the state.

That lesson is certainly relevant in the UK today, as police face declining public confidence. For a policing model built on public consent, a loss of public confidence is a crisis. Police use of force is a critical factor in that confidence. Now, more than ever, we must use it as a last resort and only when absolutely necessary.

In the UK, human rights are protected by law. Article 2 of the Human Rights Act 1998 states that ‘Everyone’s right to life shall be protected by law. No one shall be deprived of his life intentionally save in the execution of a sentence of a court following his conviction of a crime for which the penalty is provided by law’. Our training refers to the Article 2 responsibility only for the use of lethal force. Yet, I believe that omission is a mistake. The same general principle should be used for all force. In the circumstances described by Home Office statistics for England and Wales in the year ending 31 March 2021 (Home Office, 2021):

- There were 562,280 recorded incidents in which a police officer used force: 70% were perceived by the officer as White and 82% were perceived as male; 54% were perceived to be 18–34 years of age.
- Eighty-one percent of people were not perceived to have a physical or mental health condition.
- Restraint tactics (e.g. handcuffing) were the most common type of force used (80%).
- The most common reason an officer used force was to protect themselves (70%).
- The most common impact factor was the person being under the influence of alcohol (33%).
- The most common outcome was the person being arrested (76%).

When using force on members of the community, we face the danger of both over- and under-reaction in deciding what we do and how we do it. Our reaction is affected by the pressure we face from society and its politicians. In the past, we have got it wrong, sometimes willing to be swayed by both public and political opinion, despite protesting operational independence. And when we make decisions, sometimes we are too defensive, failing to understand we are not only operationally independent but also operationally responsible and must be publicly held to account. The more secret and closed we are, the more public confidence in police suffers.

So, have we learned the hard lessons of history?

It is important to understand where Sir Robert Peel and his Commissioners, Charles Rowan and Richard Mayne, stood in 1829. This trio are the real visionaries in the last near 200 years of policing. It is therefore useful to revisit the nine principles of 1829 and ask if we are living up to them in 2022.

I ask this question from the standpoint of my 30 years in policing. In my career, I have been the Senior Investigating Officer for 14 deaths in custody and 17 Trident gangland murders. I was a Gold Commander, and a senior legal client for three notorious deaths in custody. I was a Counter Terrorism Commander licenced to authorise a critical shot. I have deployed the military more than any other UK police chief outside war. I was the head of the Metropolitan Police Specialist Firearms Command during the inquiry into two fatal police shootings — Azelle Rodney^1^ and Mark Duggan.^2^ For 17 years, I was an authorising officer of the most covert and intrusive powers in policing. I was also the National Police Chiefs’ Council lead for less lethal weapons who brought the Taser X2 into the UK and repeatedly defended its increasing use. I know something about police decisions to use, and not to use, force.

So why do I ask the big question about whether we have learned the lessons of history? Why do I turn to the Nine Peelian Principles with their core idea of policing by consent? It sounds like I believe in force — I have certainly used a lot of it.

My own view was crystallised at a university dinner in Tel Aviv, attended by alumni including the former heads of the Israeli army and security services. All were combat veterans of the Arab/Israeli wars; all were arguing for a two-state solution; all were anxious not to inflict war on the next generation. Those who experience war do not want to see it repeated without deep thought. They understand that while war is the ultimate application of state force, it can only be a short-term solution. Without dialogue there will not be peace.

Our dialogue as police officers is with our public. The use of force without public trust and confidence in the people who use it, and without public consent for it, reduces our standing. Force has often been applied with dramatic consequences, but it is force plus lack of consent that can turn a drama into a crisis.

British Police training is clear. It is based on both Peelian principles and Human Rights law. It is widely and internationally respected and commands the respect of all UK Police Chiefs. It makes one thing very clear: we must think very carefully every time we want to apply force — not only the potentially devastating short-term consequences but long-term consequences as well, on community confidence, on the psychological health of the people who use it, and most importantly on the people it is used on.

The European Convention on Human Rights provides the differentiating principles between normal force and lethal force, the latter being force intended to cause lethal effect or serious injury that could lead to death. It states that lethal force must be absolutely necessary for a purpose permitted by law, the last resort, reasonable and proportionate, and the minimum required in the circumstances to achieve the lawful objective. Anything else will be seen as excessive and unlawful.

Why isn’t that the default position for all police use of force in the UK? The challenge lies in the particular facts of each difficult case. That challenge is arguably the source of the crisis of public confidence. Not since the 2011 riots, and before that the 1999 Macpherson Inquiry into the murder investigation of Stephen Lawrence, has the legitimacy of policing felt under such threat.

## Case Studies of Losing Public Confidence: a Commentary

The *Stephen Lawrence* case was not an example of inappropriate police use of force. It was a case of under-policing the murder of a young black man. Decades of post-Windrush over-policing in the black community supported the charge that British policing was institutionally racist. Our poor relationship with the black community amplified the effects of downright incompetence. It revealed to the world a disturbing police culture and a lack of trust, confidence, and consent for what we do in a community disproportionately affected by us. Thirty years later, we are still there.

The riots in London and elsewhere in Britain in 2011 were believed to be a reaction to the shooting of Mark Duggan. This was arguably not the case, as the events unfolded. The trigger instead was the consequence of the march on Tottenham police station in North London, a poor police response to the family and their supporters and their lack of trust and confidence in us.

Despite the inquest verdict, many, especially in the black community, believe Duggan’s shooting was an inappropriate use of force. I do not have that view myself. But along with many, perhaps most, police officers, I believe whenever we use force, our legitimacy is called into question. Ironically, the criticism of police reaction to the riots, not least by the Prime Minister and the Home Secretary of the day, concerned the lack of force — our invisibility and inability to restore order. My own conclusion is that if the community had trusted us, and were confident we used no more force than absolutely necessary, there would have been no march and no riot.

It is ironic that our failure to use appropriate levels of force may have been a result of policy changes caused by criticism after the death in custody of Ian Tomlinson at the 2009 G20.^3^ As a result, a different UK public order policing style was developed: community focused, operationally restrained, and based on every attempt to communicate, negotiate, and influence protest organisers. This was a very different, and arguably much better, approach to supporting lawful, peaceful protest. This was 2 years before pan-UK rioting and a new Prime Minister’s comments about the need to be more robust.

This issue is so important because the power to use coercive force rests on the police as a monopoly. Our attention to it is central. Abusing this power is a threat to policing and a common factor in many historical crises. But through those crises over the past 30 years, confidence in policing has remained at around 70%, one of the highest for any profession. But how proud can you be when 30% of the public does not trust you? The use of force always affects police legitimacy, long-term as well as short-term, and can result in trauma not only for individual but for communities as well.

The murder of Sarah Everard^4^ has led to questions about the Metropolitan Police being ‘rotten to the core’. The policing profession is now accused of institutional misogyny, in the same way that signal events have triggered accusations of institutional racism, institutional corruption, and institutional homophobia. Social media footage of the policing of Sarah Everard’s vigil was damning. It became clear that despite an IPCC report exonerating front line officers, we were tone deaf to the deeply held feelings of the community.

Within 3 months of these events, the National Police Chiefs Council (NPCC) had appointed a dedicated Deputy Chief Constable responsible for addressing violence against women and girls. It also obtained new funding for launching a refreshed national plan. This was clearly the right thing to do. But it raised questions about the far longer delay in responding equally to community concerns following the murder of George Floyd. Does this difference indicate a reluctance to accept that structural, societal, or institutional racism still exists, both by government and many of our colleagues? Does it reflect a reluctance in the face of the subjective evidence of our staff’s lived experience, and the objective statistics of disproportionality in many policies and practices such as stop and search, recruitment, promotion, retention, and misconduct?

## Apologies Made and Not Made

When we hesitate to apologise for past actions that we do not deny, it looks like wilful blindness. Our position that we have bigots, but that they are a few ‘bad apples’, an inevitable consequence of recruiting from a society with such people, is wilfully blind and tone deaf. We accept that things are so bad we need a very comprehensive plan, but we do not accept why. I am the most senior officer of colour in the UK, and I cannot live with a failure to listen to the concerns of our own hacked-off staff and marginalised communities of difference.

It is sometimes said that society gets the policing it deserves. In 1829, what did Peel think society deserved? He did not go for the French military model — copied in most countries around the world including almost all other European democracies. He went for consent built on trust and confidence. He did not give society what they wanted; historical evidence suggests they did not want policing at all. Peel gave us what we needed in the most widely admired and exported policing model in the world — policing by consent and separate from the state. It remains largely an exception: an unarmed model where the attitude to force was clear.

I always worry that the tone of society and the people who run it can affect the way we police, and that under pressure we could forget some of Peel’s key principles. When the public began to accept that policing was essential, usually on the back of a crisis, it began to accept its authority unquestioningly. Certainly, when public anxiety over the IRA was at its height, no one questioned the methods used to find the culprits of the Guildford and Birmingham atrocities. When public anxiety about street violence was at its height, no one questioned police tactics used to supress it.

It seems churlish for me to argue that having built a strong reputation from a shaky start, having won public trust and confidence to the point the public accepted and supported us, that we have lost our way. Power — and we really have such power — requires that we must constantly guard against taking it for granted and abusing it. At various points in our history, we have been remarkably complacent about that. Some officers, when given power without accountability — in other words absolute power — became absolutely corrupted by it.

In this context, the latest proposed UK legislation on protest^5^ particularly concerns me. It harks back to the 2011 riots when the Prime Minister said of the police, ‘you’re not robust enough’. Some measures *may* be necessary for effective public order policing. We should ensure they are *absolutely* necessary. We should question whether it balances majority rights with protesters rights, or whether it is designed to minimise the effect of protest, the rights of free association, assembly, speech, or the right to dissent. When we create new laws, in that moment, we may be lucky enough to live in a time with liberal democracy in which the Government is benevolent, where the security arms of the state and its policing operate under strict human rights principles. What if that changed? Are we then handing powerful laws, without appropriate safeguards, to a future, less benevolent, machine?

## Separation of Powers

Will the separation of powers hold? Will the politicisation of policing mean Chiefs under PCC or Home Office pressure, adopt increasingly robust tactics to supress dissent, when the signs are we now live in a less tolerant, more protest-hungry, and anti-authoritarian society?

In some cases, protesters may well have a point. Racial and social justice, climate change, and peace are worth fighting for. But they are less willing to listen to reasoned argument and are easily outraged by a cynically manipulated message, often delivered by that efficient propaganda machine, social media. At critical moments, someone is likely to be filming any police action, and an edited and narrow view can go viral almost instantly. We never seem ready for it. We cannot seem to defend ourselves in a highly accountable transparent world, and yet we believe in operational responsibility for our actions.

This places policing in its usual invidious position. As ever, the thin blue line stands between warring factions and also the contents and the malcontents. If we disallow protests because the contents want to get on with their lives, are we ignoring an important voice or issue? We are community leaders — sometimes in great crisis, **the** community leaders. We are guardians of society’s morality and laws. We have great power, responsibility, and discretion. The greatest power is the civil application of force, especially when depriving liberty, and on that we have a monopoly.

What we as policelack, is the cultural competence to recognise why some in our community do not trust us. We can lack the ability to reduce rather than increase the temperature of the conversation, and can’t use the art of compromise without feeling weak. We are often capable of using force instead of persuasion. Bad recruitment, vetting, and training are all part of the problem. And if we compound that by training our police officers to be fearful, force will be a first not last resort, and is unlikely to be minimal and reasonable and certainly not absolutely necessary.

My argument is that *if you want the public to trust you, the force you use must be the minimum necessary to maintain public consent*. What best describes ‘minimum’ will be contextual and unfortunately subjective. But changes in society bring about changes in police response and public acceptance of force. We can resist calls for more or less enforcement. It is our decision. But I worry that performance pressures have led us in the past to a more enforcement-oriented mind set, careless of the long-term consequences.

## Evidence-Led Use of Force Policies

Non-evidence-led business cases for increasing stop and search, without improving the skills of empathy, procedural justice, and cultural competence, are a clear mistake. The problem is compounded without data to show where, when and on whom it should be applied to be effective. It is crucial that stop and search commands public consent and is used by skilled operators who know how to speak to people. Otherwise it is just a short-term street suppression tool, one with no long-term positive effect on violence but great negative effect on community tension.

Did we notice as society’s confidence declined in both the state and policing that the confidence gaps in some communities got wider than in others? Did we ask the young what they thought? Did we pay attention to the George Floyd-like cases here, and their effect on the black community? Did we notice the consequences of immigration, demographic, and economic pressures that reinforced and multiplied difficulties in areas over-represented with people of difference, but where many distrust the Goverment and the police?

We started recording our use of force in 2017 but still we are limited by how little we know about whether it is increasing in response to increasing violence. It seems likely that the most used application of force, handcuffing, has increased when it has become a default prior to stop and search for some officers.

Stop and search is our most iconic use of force and the most visible. But do we know whether its massive increase is working? Is it reducing violent crime? If so, what proportion of violent crime’s decline can we ascribe to the use of stop and search? Is it a proportionate use of force? Is it no more than is absolutely necessary to protect society? There are so many things we do not know about such a huge subject.

**Fig. 1:**
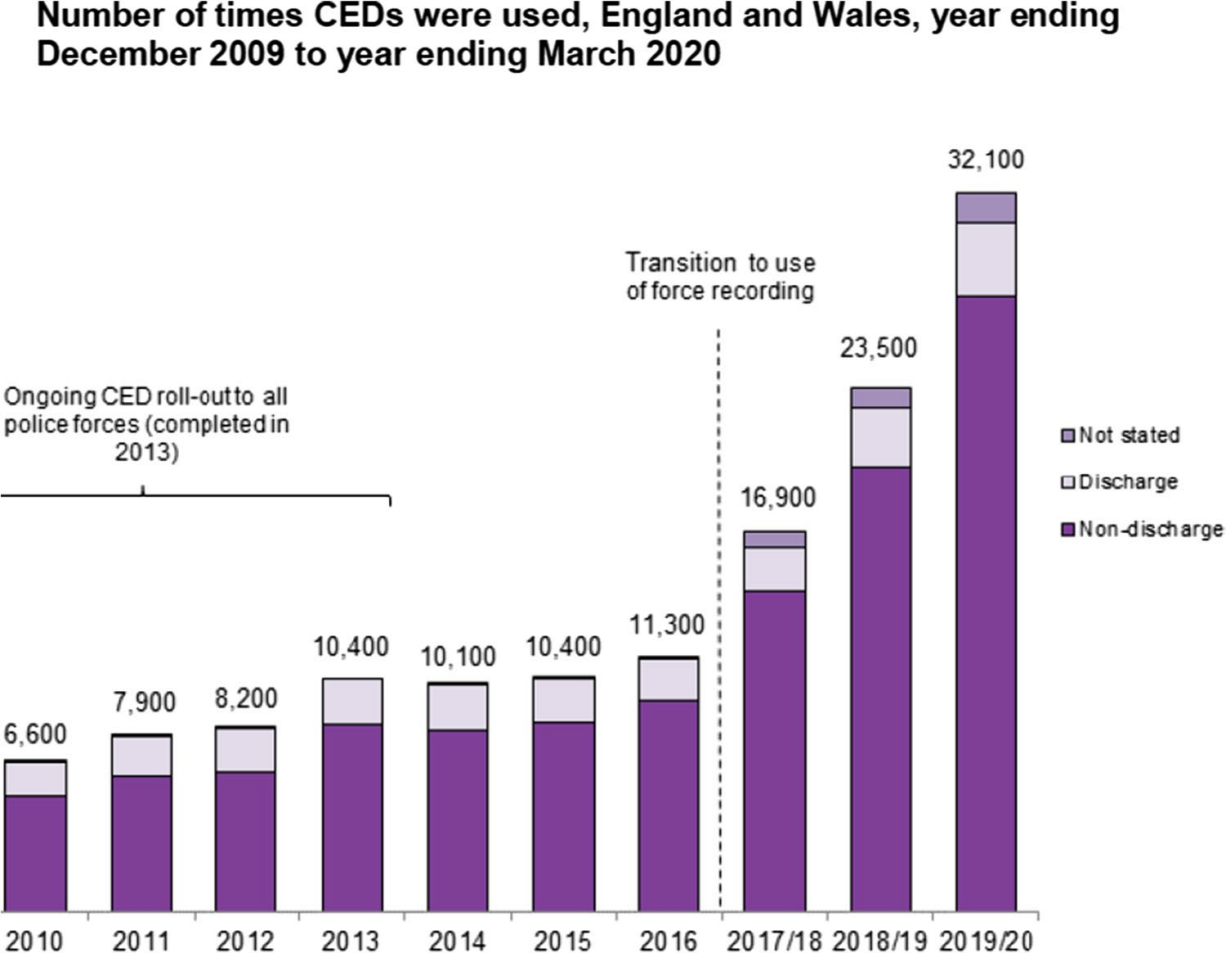
The number of times CEDs were used, England and Wales, year ending December 2009 to year ending March 2020. Reprinted from Home Office, Police use of force statistics, England and Wales, April 2019 to March 2020, Table 13; Police use of TASER® X26 conducted energy devices statistics, England and Wales, collection. Notes: CEDs were first trialled in UK police forces in 2003, after which the use of CEDs by all specially trained officers was authorised by the then Home Secretary in 2008. The CED roll out to all 43 Home Office police forces finished in 2013

Similarly, we have reacted to the increasing violence we said exists by rolling out more Tasers (Conducted Energy Devices, or CEDs) to more officers, so Taser usage is dramatically up, as Fig. 1 shows. This is our most controversial tool, with the least confidence from the black and mental health communities. Have we done the right things for the right reason based on the right data? We ought not assume citizen consent for increasingly arming the police. ‘Less lethal’ weapons and black shirts on officers create an image far removed from the traditional British bobby. No matter how professional our people, policies, and procedures, I suspect much of the public find this disconcerting.

But community policing at its best can be transformative. Even a tough operation like Trident, set up in 1998 as a community-led initiative to tackle gun crime especially in Afro-Caribbean communities in London, led to substantial reductions in murders of young black men. It could be said to owe its success to trust gained through treating the community as partners in a community that eventually invited police in. They did not want the violence, and we were effective in locking up the bad actors in their midst.

But here is a central dilemma: how to retain confidence when we are often called on to confront violence with force of our own? The public want us to be both community-engaged and enforcement-capable. But they are not sure which one of us they want and when. As the professionals, this is our problem to solve. We routinely demand both compassion and force from our police colleagues — often in the same day. We set a high bar even if it may be impossible to consistently reach.

In forensics, we use Edmund Locard’s exchange principle that ‘every contact leaves a trace.’^6^ I believe the same is true for every confrontational situation we put our officers in. And when they break, what happens? Do we adequately prepare them for this dissonance? How do we treat them afterwards? How adept are we at spotting red flag behaviour in our colleagues? Increasing coldness, a propensity to confront, a tendency to see the public as ‘them and us’? If we spot it, what tools do we give them to turn it around? If we want public support when we respond to violence with force, we need to do the groundwork with our colleagues in order to gain public trust and confidence.

## Building Public Support for Increased Use of Force

Since 2017, the UK has been rocked by multiple terrorist attacks, and increasing knife, gun, and violent robbery, and last year, London recorded the shocking statistic of thirty teenagers murdered in 1 year. How did we respond? More guns, more Tasers, more stop and search, violence suppression units, spit-hoods, more use of covert tactics, and more draconian laws for terrorism and public order. As well, we have legislation to protect emergency service workers emphasising the increasing risk they faced. This may all have been absolutely necessary. But what effort did we put into gaining community consent for it?

Lord Scarman’s definition of community policing in his 1981 report^7^ echoes Peel and remains as relevant today as then: ‘policing with the active consent and support of the community’. It is an ideal many of our police forces strive for, but this police/public partnership is a difficult balance. In 2009, Bradford et al.^8^ asked communities a straightforward question: ‘Do police do a good job in your local area?’ Based on the responses, the authors discussed the correlation between good policing and public trust and confidence. The follow-up by Jackson et al. (2012) also found that greater trust and confidence in the police and their legitimacy meant communities were more willing to report victimisation, to give intelligence, co-operate, and obey the law. All these police scholars agree there are three key dimensions affecting trust and confidence: effectiveness, fairness, and engagement, all of which we the police can influence but on which at the present time we seem to be failing.

I greatly regret how quickly, under austerity, policing in England and Wales gave up neighbourhood policing. Why was this seen as discretionary spending? How did we plan to maintain community trust and confidence to use heavy tactics when absolutely necessary, without inflaming communities? At the same time, we poured effort into high harm, threat, and risk, and away from crimes that mattered to most people — burglary, robbery, anti-social behaviour, and vehicle theft. This change made police look more remote and ineffective. With hindsight, neighbourhood policing may be the greatest police tactic ever developed, and we better put it back quickly.

Nevertheless, our job remains to prevent major disorder, solve crime, and bring offenders to justice. The public must believe we are effective as well as fair if they are to consent to our powers and particularly our ability to use force. How the public perceives policing through the lenses of effectiveness, fairness and community engagement, determines our levels of trust and confidence and nothing affects that perception more, in my opinion, than our use of force.

Alongside being approachable, the public expects us to be capable of protecting them from harm. The ability to use force — even lethal force — is intrinsic to public confidence in the police service. But when, where and ultimately how it is used is crucial. As I argued at the outset, force can never be used other than by absolute necessity. It also means that lack of confidence in use of force has to be mitigated by deep and wide community engagement before critical incidents occur.

Informing is not the same as engaging. How committed are we to actually listening and involving the community in what we do? In 2011 following the killing of Duggan, the riot may have started in Tottenham because we were not seen as legitimate. We failed to realise how little respect we had in that community. But many rioters had nothing to do with Duggan and Tottenham. We were not visible, we were not seen as effective, and youth did not respect us. I have no doubt that many did not think they would be caught.

The only thing that stopped the rioting of those 4 days in August was a massive surge in police numbers and increasingly robust tactics. Through the use of overwhelming force, we came close to losing our legitimacy for good. For many years afterwards, we continued to over-resource and over-police major demonstrations, in fear of a repeat failure. Arguably, our whole Metropolitan Police Service operating model was changed to ensure massive surge capability and sacrificed our ability to detect crime.

It is important to remember that it is not the use of force itself, even lethal force, that counts. I could talk about every time we shot a terrorist and the complete lack of public outcry. It is the context — the reason, the location, the community, the post-incident handling, and the groundwork you put in before the event happens that counts. This is what prevents a use-of-force becoming a disastrous critical incident. In communicating, we are hampered by the rigidity of our media relationships. We are hampered with our Independent Office of Police Conduct investigative protocols. We are hampered by the inflexibility of our criminal justice processes, when it comes to explaining what we have done and why. But we still have discretion.

I argue if disorder is likely, and on a massive scale, all of these factors can be overcome.

## Procedural Justice and Public Confidence

Much has been written over the past 20 years about the role of procedural justice in effective policing.^9^ Hough (2020: 7), for example, said ‘maintaining a convincing level of deterrent threat has high financial and social costs, and that securing consent to the rule of law can achieve more stable and more peaceful communities……the more that hard policing tactics are deployed, the less the police leave themselves for policing by consent’.

But along with being treated according to the law and fairly, I would add what Chief Constable Andy Marsh, Chief Executive Officer of the College of Policing, calls ‘cultural competence’: the ability to understand the person standing in front of you, the way they see the world and why. Only those with high emotional intelligence and good people skills have the best chance to de-escalate conflict and win people over. How is our training on this issue? How do we select people for possessing these skills when we recruit new officers? How quick are we to dismiss people who do not have it? How important do we think it is?

Our ability to convince the public we are on their side also depends on our corporate communications, as well as individual officer’s every interaction. How do we communicate what we are going to do or what we have done and why? How do we get that information out, so it is heard in the right places by the right people at the right time, and in language people understand?

Peel’s policing principles bind the police and the public together and provide legitimacy to the British model of policing. We must behave well and be seen to behave well to be regarded as part of the normative and ethical frameworks upon which society are based. When communities believe police no longer function as a moral authority on their behalf, this can and does lead to public disorder — a clear expression of weakened ties between police and the community.

Part of maintaining public confidence is the ability to confront the greatest possible threats, but in a balanced way. We must avoid being seen as oppressive or eroding the traditional values we place in having an unarmed service. A need for strict adherence to the law and an ethical approach of policing without fear or favour — Peelian principles themselves — have been interpreted by some that our discretion can never be used. This creates a massive dilemma for police commanders:

You can’t police a protest differently because the cause is just? Can you? Discuss!

Discuss in relation to the Everard vigil appeal? This is very dangerous ground. It smacks of politicisation. The commander will be accused of acting in a partisan way when actually they may be trying to minimise harm.

I believe our use of force is the single biggest factor in the determination of whether we will retain public confidence. Peel knew it in 1829. We better remember it in 2022. How we treat the public in the days leading up to that next major use of force will define the next generation of police leaders.

## Conclusions

In summary, here are the key points I have made:

1. The use of force is both a measure of our effectiveness and a driver of confidence.
2. Authorised Professional Practice should be studied and rigorously applied, but today, no more force than absolutely necessary should be used.
3. It is not just the level of force, but the force plus the level of public consent that counts.
4. Consent requires trust and confidence, which is driven by our effectiveness, fairness, and engagement. Broad consent is essential before force is used.
5. In a liberal democracy, the police cannot have the power to use force without accountability and transparency.
6. Operational independence to make hard policing decisions should be matched by operational responsibility and willingness to account for that decision.
7. Corporate memory of events that have undermined confidence in police is poor and so our actions are destined to repeat themselves. We have not closed the confidence gap in some communities and are wilfully blind to the reasons why.
8. Political and societal pressure can result in over- or under-reaction in the use of hard police tactics.
9. *We are not making decisions based on rigorous evidence and research, so we cannot explain why we use certain tactics and whether they are disproportionate.*
10. The evidence concerning our need for more self-protection and greater force is poor and may affect our judgement.
11. Our corporate communications are often reactive, poorly judged, and too late, affected by rules that can reduce our ability to stop disorder.
12. In summary, we need to prioritise professional standards, procedural justice, cultural competence, and empathy: our recruitment, training, and continuous professional development should focus on these.

Finally, I offer the following advice to future strategic leaders:

1. Make all use-of-force subject to an absolute necessity test, as Article 2 of the Human Rights Act (1998) requires.
2. Select recruits based on their EQ (emotional) as well as their IQ (intelligence).
3. Train police for de-escalation in conflict management.
4. Train cultural competence using history, community educators, and the young.
5. Allow communities real insight and influence in policy-making and in setting professional standards.
6. Make sure we can explain the need for our most controversial tactics based on objective evidence.
7. Be more effective in dealing with crime, fairer in the way we do it and wider and deeper in true community engagement by investing in neighbourhood, schools and faith policing.
8. Be far more proactive in the use of communications including corporate campaigns to inform who we are, what we do and why.
9. Ensure that police officers are given time and space to give back to communities they police through charitable work to avoid a ‘them and us’ mentality.
10. Improve supervision and standards for first line supervisors, reducing ratios and improving leadership training, and reconsidering single crew philosophy.
11. Be clear what the data is telling you about officer safety; how officers are at risk, when, where, and from what people and tactics — do not exaggerate the risk.
12. Ensure your officers and staff are valued by being well led, well equipped, and well cared for in mind and body so they are fit to serve their communities.

## Footnotes

1 Azelle Rodney was shot dead by a Metropolitan Police officer in April 2005. In 2013, a public enquiry found that the officer had ‘no lawful justification’ for killing Rodney; he was subsequently charged with murder but was acquitted.

2 Mark Duggan was shot dead by a Metropolitan Police officer in Tottenham, North London, on August 2011. The Metropolitan Police stated that officers were attempting to arrest Duggan on suspicion of planning an attack and that he was in possession of a handgun. The circumstances of his death resulted in public protests in Tottenham, which led to conflict with police and escalated into riots across London and other cities. An inquiry into Duggan’s death by the Independent Police Complaints Commission (IPPC) found no evidence of criminality by the police.

3 Ian Tomlinson was a newspaper vendor who collapsed and died in London after being struck by a police officer during the G-20 street protests. An inquest jury returned a verdict of unlawful killing.

4 On March 2021, Sarah Everard was kidnapped while walking home in London, raped and murdered by a Metropolitan Police officer, Wayne Couzens. Soon after his arrest, a vigil was held near where she had disappeared, leading to a controversial police response and four arrests for breaches of COVID-19 regulations.

5 Police, Crime, Sentencing and Courts Bill, 2022.

6 See https://www.sciencedirect.com/topics/social-sciences/exchange-principle, downloaded 25th May, 2022.

7 The Scarman report was commissioned following serious disorder in the mainly Afro-Caribbean community in Brixton, South London, on April 1981.

8 See also Bottoms and Tankebe (2012).

9 See for example Tankebe (2009), Bottoms and Tankebe (2012), Tyler (2003), Hough (2020).

## References

[CR1] Bottoms A, Tankebe J (2012). Beyond procedural justice: A dialogic approach to legitimacy in criminal justice. Journal of Criminal Law & Criminology.

[CR2] Bradford B, Jackson J, Stanko EA (2009). Contact and confidence: Revisiting the impact of public encounters with the police. Policing & Society.

[CR3] Home Office (2012). *Definition of Policing by Consent.* Downloaded 25th May, 2022, from https://www.gov.uk/government/publications/policing-by-consent

[CR4] Home Office (2021). *Police use of force statistics, England and Wales: April 2020 to March 2021.* Published 16 December 2021. Downloaded 25 May from https://www.gov.uk/government/statistics/police-use-of-force-statistics-england-and-wales-april-2020-to-march-2021/police-use-of-force-statistics-england-and-wales-april-2020-to-march-2021. Accessed Feb 2022

[CR5] Hough M (2020). Good policing: trust, legitimacy and authority.

[CR6] Jackson, J., Bradford, B., Stanko, B., & Hohl, K. (2012). *Just authority?: Trust in the police in England and Wales*. Willan

[CR7] Tankebe J (2009). Policing, procedural fairness and public behaviour: A review and critique. International Journal of Police Science & Management.

[CR8] Tyler TR (2003). Procedural justice, legitimacy, and the effective rule of law. Crime and Justice.

